# Saline Groundwater for Aquaculture: An Expanding Hydrogeological and Hydrogeophysical Frontier

**DOI:** 10.1111/gwat.13496

**Published:** 2025-06-09

**Authors:** Barret L. Kurylyk

**Affiliations:** ^1^ Department of Civil and Resource Engineering and Centre for Water Resources Studies Dalhousie University Halifax Canada

## Abstract

Given the chronic and multi‐faceted challenges of marine aquaculture, there is growing interest in land‐based aquaculture supported by high‐capacity saltwater wells. These wells can theoretically provide a stable, high‐quality source of saline groundwater for aquaculture tanks. In this Issue Article, I focus on saltwater wells installed in the salt wedges of coastal aquifers and argue that these wells could benefit or harm local homeowners or municipalities relying on nearby freshwater wells. More research in the fields of hydrogeophysics and physical and contaminant hydrogeology is critically needed to better understand how high‐capacity saltwater wells may impact coastal aquifers and groundwater‐dependent communities. Such work is crucial for informing the development of scientifically based regulations for the management of these wells and related aquaculture operations. Appropriate regulations would protect coastal communities, ecosystems, and industrial operators from potentially negative impacts of saltwater wells and would help to maximize their potential benefits.

## Overview

Aquaculture is a rapidly growing agri‐food sector (Tacon 2020; Verdegem et al. 2023) that is outpacing global population growth and is considered a key contributor to achieving the UN Sustainable Development Goals (Jolly et al. 2023). The global aquaculture market already exceeds 200 billion USD/year and is forecasted to exceed 350 billion USD/year within the next decade (Precedence Research 2024). However, there are many concerns regarding the deleterious impacts of marine aquaculture on coastal and marine ecosystems, including marine pollution, aesthetic impacts, and disease transfer to wild populations (Read and Fernandes 2003). Also, marine aquaculture operations are vulnerable to changing ocean conditions due to natural dynamics and anthropogenic climate change, including the impacts of marine heat waves (Calado et al. 2021). Given the challenges of marine aquaculture operations, there is increased interest in “inland saline aquaculture,” an industry term that typically refers to land‐based saltwater aquaculture operations in both inland and coastal environments (Allen and Fielder 2024). Inland saline aquaculture operations require a source of high‐quality saline water for their tanks, and water demand can be very high in large facilities (e.g., >10,000 L/s). Because coastal *surface waters* are often characterized by fluctuating temperatures and salinities and low water quality due to bacteria and viruses, saline *groundwater* offers an alternative high‐quality saline water resource with a presumably stable thermal regime, at least if sourced from below the penetration depth of transient temperature signals (Kurylyk et al. 2015). Saline groundwater can also be found far inland, including under saline lakes in endorheic, arid basins worldwide (figure 1 of Wurtsbaugh et al. 2017), and be sourced from water extracted in the coal bed methane gas industry (Allen et al. 2009). These inland sources of saline groundwater provide flexibility in inland aquaculture facility siting. Aquaculture experiments or operations supported by saltwater wells that intentionally pump saline groundwater have been active for years to decades in some countries, including Australia (Partridge and Lymbery 2008; Booth and Fielder 2016), Brazil (Espinoza Ortiz et al. 2021), Egypt (Sadek 2011), India (Barman et al. 2005), and the United States (Smith and Lawrence 1990; Forsberg et al. 1996). However, other than rare exceptions (Espinoza Ortiz et al. 2021), published studies consider operations for which wells pump saline groundwater from inland, arid basins rather than from the salt wedges of coastal aquifers. Despite the general lack of published studies on salt wedge pumping, the growing interest and recent developments in new or expanded aquaculture farms relying on saltwater wells in coastal aquifers appears to be unprecedented, at least in Canada and many European countries. These developments have prompted new government investment and the consideration of saline groundwater as a resource to be mapped and managed (e.g., Government of New Brunswick 2025). This Issue Article presents a high‐level perspective on the urgent need for more coastal hydrogeology research on this topic.

## Dynamics and Challenges of Coastal Saline Groundwater Pumping

**Figure 1:**
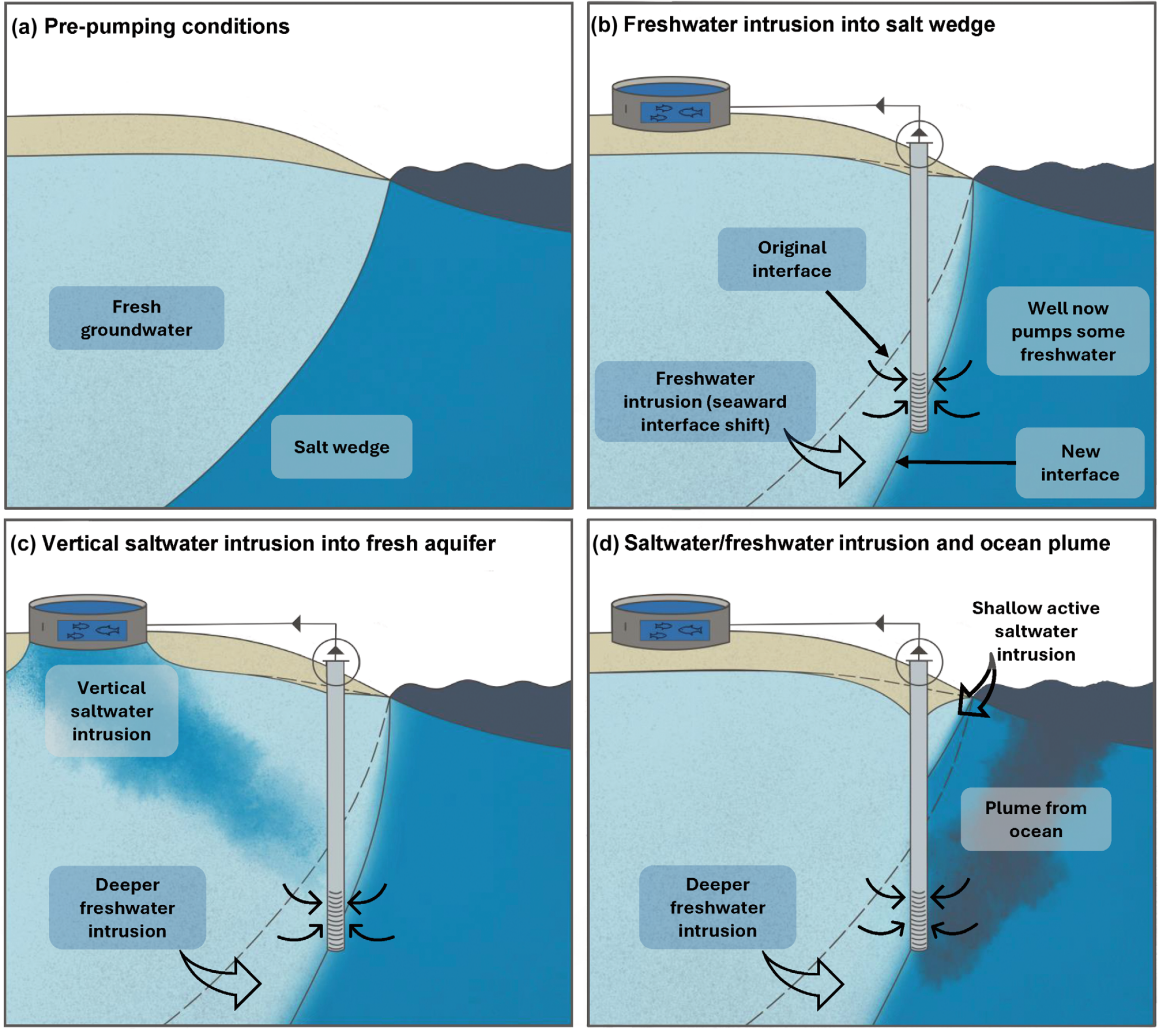
Coastal groundwater salinity distributions for (a) no saltwater well, (b) a salt wedge well with a localized cone of depression that lowers the salt wedge head and causes freshwater intrusion (interface movement in (b) is also shown in (c) and (d) to illustrate compounding effects), (c) downward saltwater intrusion from a focused source associated with an aquaculture pond or tank, and (d) similar to (b) but with a more vertically pronounced cone of depression that flips the aquifer‐ocean hydraulic gradient and triggers shallow active saltwater intrusion and a thermal or contaminant plume from the ocean (deeper freshwater intrusion depends on the cone of depression).

Recently established inland saline aquaculture facilities often target saline groundwater found in naturally occurring salt wedges of coastal aquifers (Figure 1a). In contrast with aquaculture operations in arid basins far inland, aquaculture operations that pump from coastal aquifers offer the benefit of easy access to marine ports and global markets. Hydrogeological investigations for saline groundwater resource extraction share some similarities with traditional coastal hydrogeological research and practice (e.g., Jiao and Post 2019). For example, the spatial distributions of fresh and saline groundwater before and after pumping should be mapped, the aquifer's hydraulic properties should be estimated via aquifer testing, and groundwater chemistry and microbiology should be analyzed. However, the overarching goal of pumping saline groundwater and avoiding, among other things, the intrusion of freshwater is the obverse of that for traditional fresh coastal groundwater pumping. The distinct goal necessitates a groundwater management paradigm shift because sustainability concepts for fresh groundwater pumping do not apply to saline groundwater pumping. For example, sustainability plans must consider that aquaculture operations typically circulate, rather than consume, the saline water. However, the saline discharge from an aquaculture plant may not go directly to the original source because injection wells into saline aquifers and direct routing to surface waters are both common approaches for dealing with treated plant discharge. Also, wells extracting water from the salt wedge may ultimately pull water from the ocean, as discussed more later. In this case, typical groundwater sustainability concepts and depletion concerns would not be applicable given the vast ocean volume.

Inland saline aquaculture operations that rely on salt wedge wells have the potential to benefit or deleteriously impact coastal communities and homeowners relying on relatively nearby freshwater wells. In the case of high aquifer transmissivity *T* or low storativity *S*, a saltwater well or wellfield could generate a large cone of depression that lowers groundwater levels in the freshwater zone, which could impact freshwater supply, particularly for very shallow private wells such as dug wells. Conversely, an aquifer with lower hydraulic diffusivity (lower *T*, higher *S*) has a small cone of depression radius and lower drawdown, and the drawdown could be restricted to the salt wedge area near the well. In this case, the impacts of salt wedge pumping could actually benefit coastal communities because pumping would pull the freshwater–saltwater interface seaward (Figure 1b), similar to engineered systems known as “extraction barriers” or “negative hydraulic barriers” that are implemented in coastal aquifers to prevent saltwater intrusion (Pool and Ramírez 2010). One example saltwater pumping case study with field measurements is presented by Stein et al. (2020) who discussed the impacts of salt wedge wells used to support a desalinization plant in southeast Spain. Their field and modeling results show that pumping the salt wedge resulted in coastal aquifer freshening. Also, Miller et al. (2025) recently developed a semi‐analytical solution to investigate freshwater–saltwater interface movement in response to salt wedge pumping. They showed that salt wedge pumping reduces the extent of saltwater in a coastal aquifer and that the pumping rate and well location relative to the coastline and original interface can be optimized to control the interface location.

Other complications may occur from salt wedge pumping or the operation of an inland aquaculture facility. For example, if aquaculture ponds or tanks are not lined or sealed correctly, infiltration of leaching saltwater from the aquaculture facility may cause downward saltwater intrusion into freshwater zones (Figure 1c) (Hou et al. 2022). The resultant vertical saltwater intrusion would be similar to the well‐documented, density‐dependent effects of seawater flooding on coastal aquifers (Cantelon et al. 2022; Tackley et al. 2023), except that the salinization would arise from a point source rather than a distributed source. Furthermore, if the drawdown due to salt wedge pumping is high enough, it could trigger “freshwater intrusion” (Figure 1b) or localized “freshwater downconing” into the formerly saltwater well. While this would not appear to pose a contamination risk or could even be an improvement for some purposes (e.g., drinking water as previously discussed), the freshwater intrusion would render the well useless for the intended aquaculture application. Persistent lowering of coastal groundwater heads, in the wedge or otherwise, could also drive aquifer compaction and exacerbate coastal subsidence, which is already a global problem (Herrera‐García et al. 2021) that increases local relative sea‐level rise.

High‐capacity wells may also enhance ocean‐aquifer interactions in unexpected and undesirable ways. For example, if a high‐capacity saltwater well is causing active saltwater intrusion and ultimately drawing much of the saltwater from the shallow coastal ocean (Figure 1d), high pumping rates could create plumes from the ocean that would cause the pumped saline groundwater to be at least partially characterized by the thermal (Kurylyk and Smith 2023), chemical, and microbiological regimes of coastal surface waters that are typically more dynamic than for groundwater (Figure 1d). Temperature variations and redox changes due to seawater rapidly intruding into the salt wedge may negatively impact aquaculture operations and could alter natural biogeochemical processes in coastal aquifers beyond the salt wedge. Since aquaculture operations may require species‐specific optimized temperature and salinity ranges, such dynamic salt wedge or transition zone conditions should be avoided by optimizing the well location and pumping schedules. In general, these different potential responses (Figure 1) will depend on the site operations (well placement, maximum pumping rates, pumping schedules; Miller et al. 2025) as well as the aquifer properties that control the water table slope, spatial extent of the salt wedge(s), pumping‐induced drawdown, and associated groundwater flow and transport fields.

## Lack of Regulations and Related Hydrogeological Research

I have spoken to groundwater regulators and researchers in multiple countries and have heard a recurring theme—most coastal jurisdictions do not have saltwater well regulations grounded in coastal hydrogeological knowledge. Given the noted lack of transferability of groundwater sustainability concepts for freshwater consumption and our current poor understanding of how salt wedge pumping for aquaculture may impact the levels, salinity, chemistry, and microbiology of coastal aquifers, hydrogeological studies specifically addressing this unique industrial use of coastal groundwater should be conducted. If such hydrogeological studies exist, they reside in unpublished gray literature as I could find little peer‐reviewed hydrogeological research focusing on the impacts of saltwater wells for the aquaculture industry. This paucity of hydrogeological research is in stark contrast with the abundant literature from aquaculture scientists focusing on the impacts of saltwater wells on the production and health of farmed species or other aspects of aquaculture operations. For example, a recent, comprehensive report synthesizes the scientific literature on the use of groundwater to support inland saline aquaculture (Allen and Fielder 2024) and lists 587 journal papers and reports, with most previous studies coming from India (157), Australia (116), and the United States (93) (Allen and Fielder 2024). However, a careful review of the reference list reveals that these studies overwhelmingly focus on aquaculture operations rather than the physical, chemical, and microbiological impacts of saltwater pumping on coastal groundwater resources. Furthermore, related publications are almost universally in aquaculture journals rather than in hydro(geo)ology journals. While most of these past aquaculture studies have focused on saline groundwater in inland, arid basins, there is rapidly intensifying interest in saltwater wells installed in coastal aquifers. Coastal groundwater researchers have generally not been at the table for this important topic, which is a remarkable oversight on the part of regulators and the aquaculture industry.

## Opportunities in Hydrogeology and Hydrogeophysics Research and Practice

Since hydrogeologists remain conspicuous by their absence on this research topic, there are abundant hydrogeology *research* opportunities that could support related opportunities in hydrogeological *practice*. There is a long history of hydrogeology researchers contributing to our understanding of coastal groundwater dynamics, as evidenced by the biannual Salt‐Water Intrusion Meetings (SWIM, http://www.swim‐site.nl/) that have been running since 1968, as well as the Coastal Groundwater Network of the International Association of Hydrogeologists (https://cgn.iah.org/).

One primary research opportunity on this topic is large‐ and local‐scale mapping of zones of fresh, saline, and transitional‐salinity groundwater. ‘Prospecting’ for brackish or saline groundwater could be informed by well measurements but is also an opportunity for hydrogeophysics, including airborne electromagnetic geophysics for large‐scale mapping (e.g., Attia and Tsai 2024; Hingst et al. 2024). Understanding where salt wedges are present onshore or offshore (due to confined conditions) and the geometry of such wedges is critical for regulators and other decision makers evaluating the possibility to scale up groundwater‐based aquaculture operations to contribute to local economies. Depending on the species in question, aquaculture operations may wish to target transitional zones between fresh and saline groundwater, but these are known to be vulnerable to proximal groundwater pumping, are challenging to monitor (Levanon et al. 2013), and likely require more research and regulatory attention. Once these groundwater salinity zones are mapped, the aquifer properties (e.g., *S*, *T*) should be estimated through pumping tests or through analyses of tidal groundwater level oscillations (Zhang et al. 2021). Ongoing aquifer monitoring in fresh and saline groundwater zones should consider groundwater levels and salinity and should include other groundwater quality testing, particularly if landward flow of seawater is a concern (Figure 1d).

Coastal aquifer characterization and geophysics‐based delineation of groundwater salinity zones are invaluable for calibrating numerical models of variable‐density groundwater flow and coupled solute transport (Pavlovskii et al. 2022). Such calibrated models are critical for informing monitoring protocol and assessing the potential impacts of saltwater well placement, pumping schedules, and injection of aquaculture facility discharge. Reactive transport modeling should also be conducted to consider the potential impacts of changing temperature, dissolved oxygen, and other biogeochemical conditions in coastal groundwater systems triggered by high rates of saltwater pumping. Contaminant transport models could also be applied to investigate alternative options for the plant discharge, including potentially costly injection wells or via direct discharge back to coastal surface waters, which may create thermal, chemical, or microbiological surface water quality issues. Once enough site‐specific field and modeling studies have been completed, regulatory and technical guidelines could be developed based on local hydrogeological conditions.

In summary, global aquaculture production is expanding rapidly, and there is intensifying interest in the development of saltwater wells in coastal aquifers to support land‐based aquaculture operations. Hydrogeology is an applied scientific discipline that has long supported critical industries, including petroleum and agriculture. However, the rapid growth of inland aquaculture operations reliant on saltwater wells has been an often overlooked application of hydrogeological research and practice. Moving forward, coastal hydrogeological studies should be conducted to ensure related operations are sustainable and not detrimental to the society they purport to support.

## Author's Note

The author does not have any conflicts of interest or financial disclosures to report.

## Data Availability

Data sharing is not applicable to this article as no new data were created or analyzed in this study.
